# Using local gene expression similarities to discover regulatory binding site modules

**DOI:** 10.1186/1471-2105-7-505

**Published:** 2006-11-17

**Authors:** Bartek Wilczyński, Torgeir R Hvidsten, Andriy Kryshtafovych, Jerzy Tiuryn, Jan Komorowski, Krzysztof Fidelis

**Affiliations:** 1 lnstitute of Mathematics, Polish Academy of Sciences, Warsaw, Poland; 2 Linnaeus Centre for Bioinformatics, Uppsala, Sweden; 3 Genome Center, UC Davis, Davis, CA, USA; 4 Institute of Informatics, Warsaw University, Warsaw, Poland

## Abstract

**Background:**

We present an approach designed to identify gene regulation patterns using sequence and expression data collected for *Saccharomyces cerevisae*. Our main goal is to relate the combinations of transcription factor binding sites (also referred to as binding site modules) identified in gene promoters to the expression of these genes. The novel aspects include local expression similarity clustering and an exact IF-THEN rule inference algorithm. We also provide a method of rule generalization to include genes with unknown expression profiles.

**Results:**

We have implemented the proposed framework and tested it on publicly available datasets from yeast *S. cerevisae*. The testing procedure consists of thorough statistical analyses of the groups of genes matching the rules we infer from expression data against known sets of co-regulated genes. For this purpose we have used published ChIP-Chip data and Gene Ontology annotations. In order to make these tests more objective we compare our results with recently published similar studies.

**Conclusion:**

Results we obtain show that local expression similarity clustering greatly enhances overall quality of the derived rules, both in terms of enrichment of Gene Ontology functional annotation and coherence with ChIP-Chip binding data. Our approach thus provides reliable hypotheses on co-regulation that can be experimentally verified. An important feature of the method is its reliance only on widely accessible sequence and expression data. The same procedure can be easily applied to other microbial organisms.

## Background

As the shape of an organism's transcriptome is determined not only by the coding sequences of the genome but also by the mechanisms of gene regulation, massive efforts in sequencing require a similar follow up with the analysis of regulation systems. In recent years, technology has provided powerful tools to address this task, allowing for the accumulation of sequence, microarray, and functional data. Similar advancements are needed in the computational methods of data analysis.

The key role in transcriptional regulation of genes is played by a group of proteins called transcription factors (referred to as TFs) [1,2]. Their main function is to bind to the DNA upstream of a gene and take part in initiating transcription. TFs bind to the upstream DNA sequence selectively, i.e. TFs recognize specific DNA sequence motifs. Another important property of TFs is that they often interact with each other to create functional protein complexes [2,3]. Finding the connections between TFs as well as their respective binding sites and understanding the combinatorial nature of their interactions is currently an active field of research.

Our method aims at discovering binding site modules, i.e. functional sets of binding sites present in upstream regulatory regions of genes and used by several TFs in combination to regulate the expression of these genes. This task presents a considerable challenge because motif data are usually derived only from statistically significant over-representations of hypothetical binding sequences and therefore contain many false positives (i.e. occurrences of motifs that are not active in the cell *in vivo*). Also, a significant amount of noise in the expression data may lead to errors in identifying correlations among genes.

Several studies [3-7] have applied gene expression clustering to find groups of genes that may be co-regulated. However, some of the transcription factors are active only during certain parts of the cell cycle [7], and therefore genes that are co-regulated by those factors show strong expression correlations only over a subset of data points. Consequently, these modules may sometimes be undetectable by means of global expression clustering. Here we show how local expression clustering may overcome these shortcomings by means of an extensive comparison of the performance of local and global expression similarity in detecting biologically significant binding site modules.

### Approach

Our method identifies regulatory binding site modules by means of analyzing their ability to predict gene expression. To this end we systematically examine all the combinations of binding sites suggested in the Hughes study [8]. As input data we use the expression time-profiles of a large group of genes, and information on the presence or absence of binding site motifs in the promoters of all genes in the studied genome. Genome-wide expression data are readily available for many organisms, for example from public databases such as ArrayExpress [9] or Stanford Microarray Database [10]. As for motif data, there are extensive databases such as Transfac [11] covering experimentally verified motifs for many model organisms. In cases where there is not enough motif data, putative motifs may be obtained using one of the well established motif finding tools (e.g. [12-20]).

In the present study we expand on the framework we have previously developed [4] by adding a capacity for analyzing local expression similarity. Furthermore, instead of the heuristic algorithm we have used earlier, we perform an exact and exhaustive search of the rule space. We strive for improvements in both sensitivity and coverage of our approach.

**Figure 1:**
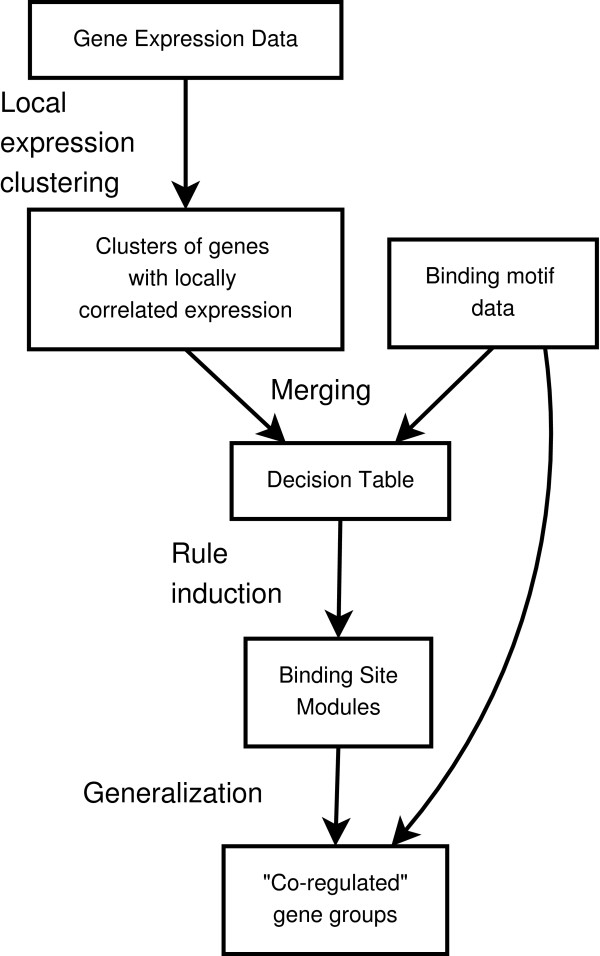
Methodology.

**Figure 2:**
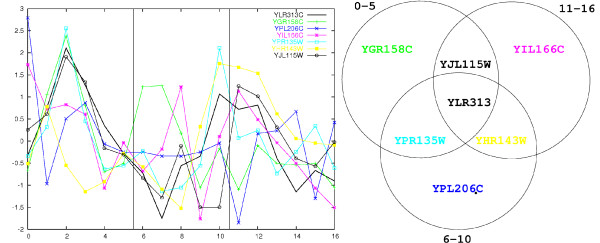
**Local gene correlations example**. Example of Local gene correlations. The plot to the left shows the expression profiles of 7 genes during the cell cycle (data from Cho et al. [29]). Three different time windows are marked by vertical lines. The Venn diagram to the right assigns the genes to clusters of co-expressed genes (correlation coefficient > 0.7) for the respective time windows. Although there are some visible correlations in all three time windows, these similarities cannot be well described by means of global correlation. Introducing analysis of local similarity allows for a better gene clustering, as shown in the Venn diagram.

**Figure 3:**
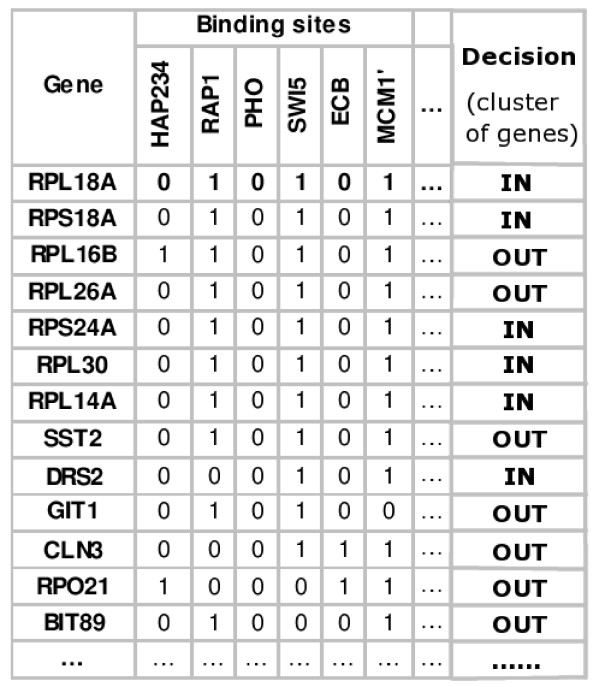
**Sample decision table**. Sample decision table taken from [4]: genes are represented as rows with the cluster membership marked in the last column as a decision class attribute.

The overall methodology of the current study is schematically depicted in Figure 1 and consists of four steps (described in detail in the following sections and in Methods):

• **Clustering**. Selecting all groups of genes that are co-expressed (in terms of Pearson correlation) limited to any sufficiently long period of time (for an example see Figure 2).

• **Merging**. Combining motif data and clustering information into decision tables, one table per cluster (as in Figure 3), such that each cluster is a decision class in the respective table.

• **Rule induction**. Finding all motif combinations capable of predicting the expression behavior of each cluster.

• **Generalization**. Assigning genes that lack expression data to modules based on the presence of significant motifs in their promoters.

#### Local correlations

We assume that genes that are co-regulated by common transcription factors display strong correlation in their expression profiles. Some transcription factors, however, may bind only under certain conditions. Indeed, many genes from *S. cerevisae *demonstrate expression correlation only during parts of the cell cycle (see Figure 2). For these genes it is not possible to obtain clusters reflecting this property by using expression similarity over the entire expression profile. However, by introducing time windows and allowing for cluster overlaps, it is possible to group genes into clusters with tightly correlated local expression. The advantage of local expression correlations was earlier identified by Gasch et al. [7], who used a fuzzy k-means clustering to allow for multiple cluster assignments of some genes. Their method however was not designed to infer temporal properties of co-regulation. Time window analysis of expression profiles was then introduced by Lagreid et al. [21]. The authors used expression analysis and machine learning methods to predict gene function (specifically GO biological process annotation of genes).

The notion of local analysis of expression profiles leads to a modified approach to profile clustering. We consider groups of genes that show very high correlation throughout a part of the expression profile. Specifically, we take into account all possible time spans of a given length (in our case ranging from 4 to 8 consecutive time points) and using a simple clustering algorithm (see Methods) we obtain all groups of genes with expression profiles correlated in any time window.

#### Merging expression and motif data

Once we compute all sets of locally co-expressed genes we relate that information to motif data. For each cluster, we create a decision table as shown in Figure 3 with genes from the cluster labeled IN and all other genes labeled OUT. In these tables, rows correspond to genes and columns correspond to binding site motifs. If a motif is present in the promoter of a gene, then we place a value of 1 in the proper row and column (otherwise the values are set to 0). The last column is the decision class, containing the value *I N *if the respective gene is a member of the considered cluster (or *OUT *otherwise).

#### Rule induction

As a learning model for describing putative regulatory binding site modules, we develop implication rules of the following form:

RAP1∧SWI5∧MCM1′→[2...10]∼(0.9)YLR197W
 MathType@MTEF@5@5@+=feaafiart1ev1aaatCvAUfKttLearuWrP9MDH5MBPbIqV92AaeXatLxBI9gBaebbnrfifHhDYfgasaacH8akY=wiFfYdH8Gipec8Eeeu0xXdbba9frFj0=OqFfea0dXdd9vqai=hGuQ8kuc9pgc9s8qqaq=dirpe0xb9q8qiLsFr0=vr0=vr0dc8meaabaqaciaacaGaaeqabaqabeGadaaakeaacqWGsbGucqWGbbqqcqWGqbaucqaIXaqmcqGHNis2cqWGtbWucqWGxbWvcqWGjbqscqaI1aqncqGHNis2cqWGnbqtcqWGdbWqcqWGnbqtcuaIXaqmgaqbamaaoqcaleaacqGGBbWwcqaIYaGmcqGGUaGlcqGGUaGlcqGGUaGlcqaIXaqmcqaIWaamcqGGDbqxcqWI8iIocqGGOaakcqaIWaamcqGGUaGlcqaI5aqocqGGPaqkaeqakiaawkziaiabdMfazjabdYeamjabdkfasjabigdaXiabiMda5iabiEda3iabdEfaxbaa@5410@

The left-hand side (LHS) of the rule requires a set of motifs to be present in the upstream regulatory region of a gene. The right-hand side (RHS) predicts that the expression of that gene over some data points (e.g. from second to tenth) will be similar (e.g. with correlation coefficient > 0.9) to the expression profile of the cluster representative (e.g. YLR197W). We say that a gene *matches *the left-hand side of the rule if its promoter contains all the motifs enlisted in that rule. Similarly, we state that a gene matches the right-hand side of the rule if its expression profile in the proper time window is correlated with the expression of the cluster representative with a coefficient greater than the given threshold.

We cannot claim that the rules we derive fully describe the complex nature of gene regulation. Rather, we aim at providing a simple model linking the available types of data, which oftentimes are marred by considerable levels of noise. Therefore, instead of interpreting the rules in the strict sense (i.e. the left-hand side matching the right-hand side exactly, with genes containing the specified motifs in their promoters forming the corresponding expression profile cluster), we look for rules satisfying two criteria we have introduced previously [4]:

• **generality**, i.e. each rule covers at least 5 genes,

• **accuracy**, i.e. of all the genes matching the left-hand side of the rule, at least 2/3 of them match the right-hand side of the same rule.

For the task of rule induction, we use an exact algorithm based on suffix trees (see Methods).

#### Rule generalization

We are interested in finding out whether the rules obtained from the procedure described in the previous section can be used to generate sound hypotheses on co-regulation of genes of unknown expression, i.e. could the information contained in the rules be extended to genes not included in the expression dataset? We investigate this question by searching for the previously identified binding site modules in the promoters of genes with unknown expression, and as a result create larger sets of putatively co-regulated genes. We can then evaluate these extended gene sets and compare with previous results.

## Results

We have tested our methodology on a cell cycle expression data set from *S. cerevisae *(see Data Material). Using a range of time windows (from 4 to 8 time points) and correlation thresholds (from 0.6 to 0.9) we obtained 3446 rules describing 337 unique binding site modules (several rules specifying the same binding site module were obtained from different time windows). These rules describe the regulation of approximately 1500 genes of the *S. cerevisae *genome. Even though our input dataset corresponds to the best-understood biological process of one of the most extensively described species, the knowledge of underlying biological processes is far from sufficient to enable direct evaluation of our rules. Indeed one would be hard pressed to find many reported examples of the effects of a particular combination of TFs on a given gene measured under specific conditions in the cell. Instead, we limit our evaluation to genomic-scale type comparisons with the available experimentally-derived datasets on gene regulation in *S. cerevisae*. We provide an extensive analysis of the statistical significance of our results in comparison with published studies concerning *S. cerevisae *cell cycle datasets. We also present two examples showing how rules obtained for particular time segments of the cell cycle can provide a more specific description of regulation: rules that could not be discovered without current improvements.

To assess the statistical significance of our results, we use the scoring procedure introduced in our earlier work [4]. We test putatively co-regulated groups of genes for overrepresentation of annotations from Gene Ontology [22] and the actual binding of transcription factors in *S. cerevisae *[23] (details of the scoring methodology are described in the following sections). To allow for a more direct and complete comparison with the results of other methods [4,24-26] we compute our scores for the groups of putatively co-regulated genes they provide. This was also done in an attempt to put all the available results on the same footing with respect to changes in GO annotations over time and with respect to the available experimental binding data. Although all authors use very similar methods of measuring statistical significance, we fully realize that the above may not be the only way to make such comparisons [27].

In the case of our own approach, evaluation with experimental binding data surprisingly produces better results for groups supplemented with genes not included in the expression dataset. We investigate this phenomenon further by using a second type of evaluation, a pairwise co-regulation coefficient (PCC). PCC is characterized by a different dependency on cluster size than the p-value based scores and thus provides an alternative way to evaluate results. The outcome of this evaluation procedure is presented in a separate section.

### Evaluation with Gene Ontology

Genes sharing a common function are very often co-regulated. Many authors use the publicly available information on genes sharing function to assess their predictions of co-regulation [4,6,24-26]. Although very indirect, this may serve as a crude estimate of the biological significance of the rules we derive. Following this line of reasoning we use Gene Ontology [22], a human-curated set of biological terms (referenced to as GO terms) to annotate the function of genes. The terms are organized into three hierarchies (molecular function, cellular component and biological process) describing biological roles at different levels of detail.

In this work we employ the scoring procedure from Hvidsten et al. [4] introduced by Cho et al. [28]. This procedure treats all sets of genes sharing common Gene Ontology annotations as sets of co-regulated genes. To this end we consider all GO terms at all levels in each hierarchy and treat all genes annotated with that term and its descendants as sharing annotation (see Methods for details).

Gene Ontology scores for our sets of rules (one set for each expression correlation threshold) are summarized in Table 1 and are compared with scores for previously published studies.

**Table 1:** Evaluation with Gene Ontology

Study	Molec. function	Biol. Process	Cell. comp.
Previously published studies			

Segal et al. [26], Cell Cycle, 17 modules	0.353	0.353	0.471
Segal et al. [25], 48 modules	0.250	0.417	0.312
Beer et al. [24] 49 modules	0.426	0.617	0.583
Hvidsten et al. [4] Cell cycle 109 rules	0.308	0.462	0.410

Present method			

corr. 0.6, 1316 rules	0.335 (0.349)	0.479 (0.510)	0.422 (0.406)
corr. 0.7, 807 rules	**0.522 **(0.395)	**0.657 **(0.532)	**0.621 **(0.551)
corr. 0.75, 643 rules	**0.453 **(**0.443**)	0.526 (0.471)	**0.711 **(0.480)
corr. 0.8, 487 rules	**0.427 **(0.361)	0.491 (0.304)	**0.719 **(0.476)

Comparison of the Gene Ontology-based scores with other studies. For each method, the presented number represents the fraction of significant gene groups among all gene groups provided by that method (significance is defined as Bonferroni corrected p-value being below 0.01). For the present method, the results that score higher than all other studies are shown in bold. Correlation thresholds used in the clustering procedure are shown in the left column. Numbers in parentheses denote scores obtained for all genes (i.e. both genes with and without expression profiles).

### Evaluation with experimental binding data

In the case of *S. cerevisae*, there is a much more direct way to evaluate results than using Gene Ontology. Harbison et al. [23] published the results of ChIP-Chip experiments measuring p-values of the binding of 352 transcription factors to 6229 intergenic regions. For evaluation we consider genes with promoters that are bound by the same transcription factor as co-regulated by that TF (see Methods for details).

Table 2 presents the fractions of gene groups scored as significant. It is especially intriguing how greatly the scores are improved by the addition of genes without assigned expression profiles. The latter is generally not the case when evaluations are performed with GO. For comparison, in Table 2 we include the scores of other studies computed in the same manner.

**Table 2:** Evaluation with experimental binding data

Previous studies	binding score
Segal et al. [26] Cell Cycle, 17 modules	0.588
Segal et al. [25] 48 modules	0.271
Beer at al. [24] 49 modules	0.286
Hvidsten et al. [4] Cell cycle 109 rules	0.538

Present method	binding score

corr. 0.6, 1316 rules	0.445 (**0.776**)
corr. 0.7, 807 rules	**0.643 **(**0.844**)
corr. 0.75, 643 rules	**0.605 **(**0.850**)
corr. 0.8, 487 rules	**0.700 **(**0.879**)

Comparison of binding scores (data from Harbison et al. [23]). For each method, the presented number represents the fraction of significant gene groups among all gene groups provided by that method (significance is defined as Bonferroni corrected p-value being below 0.01). For the present method, the results that score higher than all other studies are shown in bold. Correlation thresholds used in the present clustering procedure are shown in the left column. Numbers in parentheses denote scores obtained for all genes (i.e. both genes with or without expression profiles).

One has to keep in mind that although ChIP-Chip data currently represents the most direct measurements of transcriptional regulation on a genomic scale, it is only a statistical measure of the TFs binding to promoter regions under some specific conditions. In reality, transcriptional regulation is a time-dependant process, with TFs dynamically binding and unbinding to DNA, and cannot be accurately described by a single "snapshot" as in ChIP-Chip data. Nonetheless we accept this simplification since no better data currently exist. It would be very interesting to include time-series ChIP-Chip data in our analysis once they become available.

### Pairwise Co-regulation Coefficient (PCC)

Since our scoring is based on statistical significance of gene group overlap, it can be argued that the increase in the scores of generalized rules can be attributed to the increase in gene group size rather than to a better quality of rules. To investigate this, we need a different scoring methodology providing additional insight into rule quality for large groups of genes. To this aim we employ the pairwise co-regulation coefficient (PCC). For a given set of gene groups the PCC measures the fraction of gene pairs from the same group that are actually co-regulated (according to data from Harbison et al. [23], *p *< 0.01). Here, we choose to restrict our analysis to the experimental binding data as it provides a much more direct way of evaluating co-regulation than the GO annotation enrichment.

A PCC value can then be interpreted as follows: Assuming that we choose a random pair of genes annotated as co-regulated according to some prediction method (i.e. assigned to the same group of "putatively co-regulated" genes), what are the chances that they are actually co-regulated (i.e. have an experimentally verified common regulator)? Moreover, the PCC value has the nice property that all TFs that bind to at least two genes in the same module contribute to the score, and hence all possible subgroups of co-regulated genes are accounted for in one value.

Since the number of pairs increases proportionally to the square of the number of genes it is less likely for a large group to obtain a high PCC score than for a small one. This is due to the fact that the introduction of a new gene which is not co-regulated with the others to a group of size *n *will introduce *n *erroneous pairs that have to be accounted for in the score.

The results of this type of analysis and comparison with other studies are summarized in Table 3. To provide reference values for our results we have computed the PCC scores also for random sets of genes. We sampled 3 families of random gene sets generated to reflect the size and number of sets in the real data with the following results:

**Table 3:** PCC evaluation

Previous studies	PCC
Segal et al. [26] Cell Cycle, 17 modules	0.365
Segal et al. [25] 48 modules	0.152
Beer et al. [24] 49 modules	0.182
Hvidsten et al. [4] Cell cycle	0.353

Present method	PCC

corr. 0.6, 1316 rules	0.359 (0.383)
corr. 0.7, 807 rules	0.458 (0.452)
corr. 0.75, 643 rules	0.496 (0.479)
corr. 0.8, 487 rules	0.545 (0.505)

Comparison of pairwise co-regulation coefficient for rules obtained in this study with gene groups from other studies (values for generalized rules are shown in brackets). Correlation thresholds used in our clustering procedure are shown in the left column.

• random sets of genes sharing common binding motifs (but not necessarily co-expressed) (*PCC *= 0.171, st.dev = 0.062),

• random sets of co-expressed genes (not necessarily sharing common binding motifs) (*PCC *= 0.109, st.dev. = 0.020),

• totally random sets of genes (*PCC *= 0.020, st.dev. = 0.019).

The PCC scores for our approach show that the increase in the size of the groups caused by the generalization of rules does not result in a notable change in the quality of the rules. Although this indicates that the surprising increase in p-value significance (Table 2) is largely due an increase in the number of genes for which the statistical test is conducted, the PCC scores indicate that generalization indeed can be used as a tool for predicting co-regulation of genes with unknown expression.

### Predictive strength

**Figure 4:**
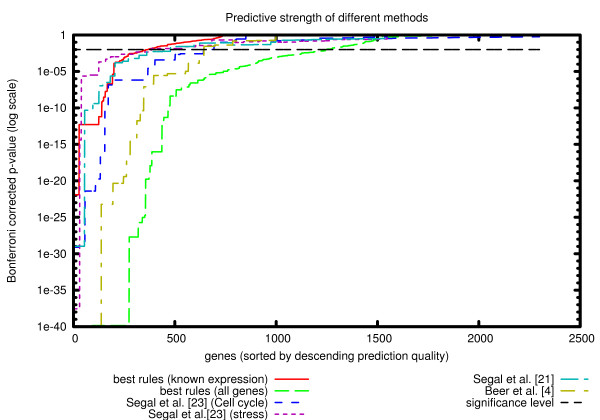
**Predictive strength**. Predictive strength. Comparison of the predictive strength of our method with other approaches. Each study is represented by a line. Along the x-axis genes are ordered by their respective p-values and these p-values are shown on the y-axis (log scale). Genes that are not characterized by a given method are omitted from the plot.

**Figure 5:**
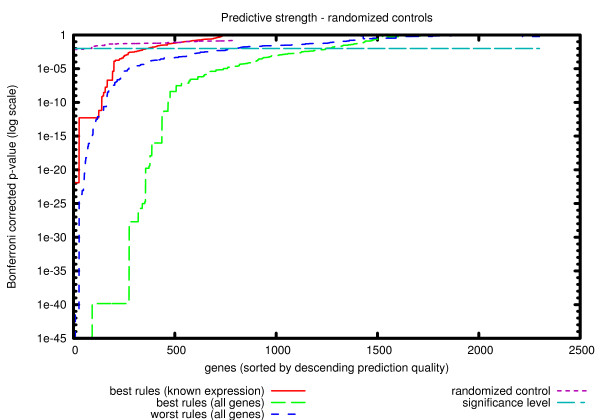
**Predictive strength – control experiments**. Predictive strength – control experiments. Comparison of the predictive strength of our method with control data. Each calculation is represented by a line. Along the x-axis genes are ordered by their respective p-values and these p-values are shown on the y-axis (log scale). Genes not characterized are omitted from the plot.

To quantify the predictive value of the system of rules as a whole, we have to measure the quality of the rules for all genes. Towards this aim we use the experimental binding data and compute the p-values of all rules as in the previous section. As a result of this procedure we obtain p-values for all the genes covered by the rules. Since the results of different studies cover different sets of genes, we have decided to present the data as a plot (see Figure 4), with the Y-axis representing p-values, and the X-axis all the genes sorted by the best p-value. We use the logarithmic scale in Y to emphasize the region around the significance threshold (0.01). The results for each method are represented by a line with each point (*x*, *y*) representing the number of genes (*x*) covered by the rules with a p-value of *y *or lower (i.e. more significant). If a gene is assigned to one rule only, the respective p-value can be interpreted as a predictive strength of the method for this particular gene. Because our method describes regulation in terms of overlapping modules it is more difficult, compared to the other approaches, to associate each gene with only one p-value. To demonstrate the full potential of the method we construct the plot in Fig. 4 using p-values of the best scoring rules for each gene. As a control we also show a similar plot using the p-values of the worst scoring rules (Fig. 5).

Given a set of rules, an attempt can be made at predicting regulation patterns for other genes in the system, in particular the ones for which no expression data are available. A simple procedure to follow is to select the rules matching the gene in question. Since our rules are labeled with binding site modules and expression profiles, they provide a hypothetical regulatory circuitry for that gene. A similar approach could be applied to results of other studies; however, not all of them mark their groups of putatively co-regulated genes with motifs and expression profiles.

We can see from the plot in Figure 4 that although our methodology (using rule generalization) does not have the highest coverage of all studies in general, it clearly performs best in the area of significant p-values (i.e. it covers more than 1200 genes with p-value < 0.01 whereas other methods cover less than 600 with the same p-value). It is important to note that if we do not include the genes with unknown expression in the analysis, the predictive value of our rules is reduced to a limited set of genes, comparable in size to those obtained with other methods (~300 genes with p-value < 0.01).

Since our method provides significantly more gene groups (i.e. rules) that other methods do, it may be argued that the real reason behind the high coverage we obtain is the large number of overlapping rules and the fact that we select the best rule for each gene. To this end we compare our results with two additional plots (in Figure 5):

• Scores for the set of generalized rules, calculated by taking the worst rule instead of the best rule for each gene. As we can see from the figure, even if we deliberately choose the worst rule for all genes, we can still annotate over 600 genes with rules that are significant.

• Scores for the set of generalized rules calculated by taking the best rule for each gene after randomly permuting all gene labels. This test clearly shows that even if we consider a large number of overlapping rules and select the best rules for each gene, we should not expect many genes to be covered by significant rules by chance.

### Specific examples

In addition to genome wide analysis, it is interesting to take a look at some specific examples of rules, to examine if the information they provide is indeed biologically meaningful. In this paragraph we examine two putative binding modules.

**Figure 6:**
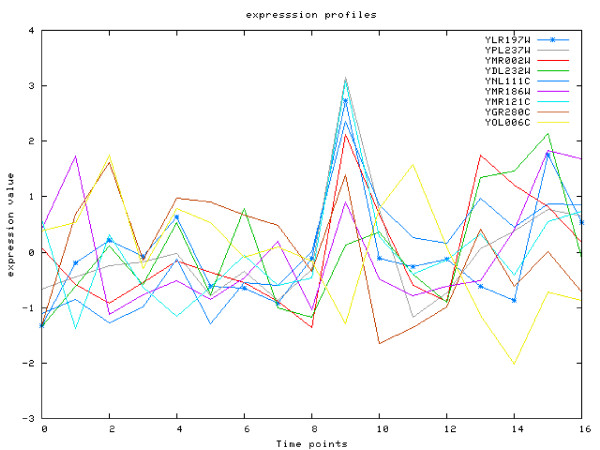
**Case Study 1**. Case Study 1. Expression profiles for genes targeted by REB1, SWI5 and SCB. It can be seen that, despite weak global correlation, expression profiles correlate locally around time point no. 10, corresponding exactly to the M1/G transition phase boundary – the activation time of SWI5.

The first combination of motifs, **REB1**, **SWI5 **and **SCB**, can be found overall in 19 rules, all of which are based on time windows comprising the 9th and 10th data points. Based on the original publication of the expression profiles [29] we can map these points to the M1/G phase boundary which is exactly the active time of the SWI5 factor (according to Saccharomyces Genome Database). It is interesting that this combination of motifs was not identified by our previous study [4]. Such identification was not really possible, since the expression profile similarity is strong enough only in the part around the 10th time point (see Figure 6). This particular combination of motifs is also strongly supported by a highly significant p-value of 4 · 10^-6 ^for the ChIP-Chip data.

**Figure 7:**
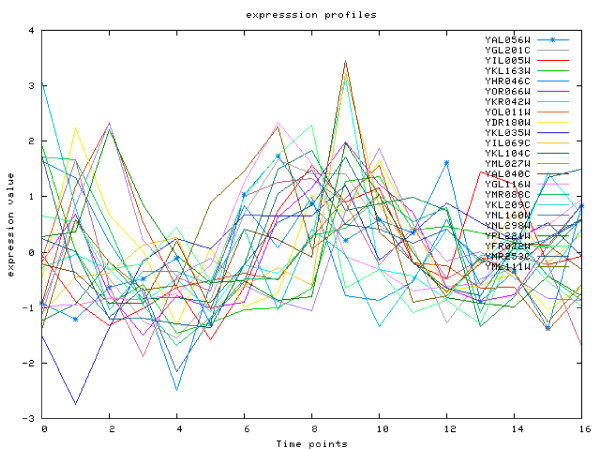
**Case Study 2**. Case Study 2. Expression profiles for genes targeted by MCM1, SWI5 and ECB. Despite no global correlation of expression profiles, the clustering detects the increasing trend in expression over timepoints 3–10, which can be attributed to activity of the ECB-binding protein.

Another interesting example combines motifs **MCM1**, **SWI5 **and **ECB**. This binding module is also not found in the previous study, where only combinations of MCM1' with SWI5 are reported. Again the reason behind missing this combination in the earlier work is the local nature of the expression correlation that cannot be identified in a global analysis (see Figure 7). However, applying local clustering identifies this module as significant in 71 rules, all of which are based only on time windows starting at the 2nd, 3rd or 4th time point. This is reasonable, since the ECB motif denotes early cell-cycle box. The module is also highly significant in terms of binding evaluation: *p *= 1.49·10^-8^.

The two examples presented here show that our approach based on local expression similarity can retrieve significant binding site modules that cannot be found by other methods based on global similarity. Furthermore, these modules were not only shown to be statistically significant (i.e. *p *< 10^-6 ^using experimental binding data), but also biologically significant (i.e. verified by published experiments).

## Discussion and conclusion

Recently, several studies have investigated the combinatorial nature of gene regulation. By analyzing expression coherence of genes containing pairs of binding site motifs in their promoters, Pilpel et al. [3] demonstrated that combination of binding sites provides a key to understanding regulation. Several later studies [24-26] employed the methodology of Bayesian networks to investigate the structure of regulatory combinations in *S. cerevisae *using expression data and promoter sequences. The aim of those studies was to identify a partition of the *S. cerevisae *genome into non-overlapping clusters of putatively co-regulated genes. Segal et al. [26] proposed using Bayesian models, where genes were assigned to clusters based on the presence of sets of motifs in their promoters. The authors employed the expectation maximization (EM) learning procedure to build a model that best matched the data. During optimization, both gene and motif sets were changed until convergence. As a result the authors obtained a rich probabilistic model describing combinatorial regulation. Beer and Tavazoie [24] took a slightly different approach by fixing the clustering and learning a Bayesian network to predict expression profiles. Again, a very complex model taking into account motif placement and orientation was obtained. However, by fixing the clustering (and removing clusters too small for accurate prediction) the authors decreased the number of model parameters which lead to better convergence of the learning process.

Previously [4] we have proposed a change to this paradigm. The idea was to induce rules connecting groups of binding sites (so-called binding site modules) with possibly overlapping clusters of genes characterized by similar expression profiles. The method aimed at finding general rules predicting similarity in expression from the occurrence of sequence motifs. Groups of putatively co-regulated genes could then be obtained by selecting genes matching the left-hand-side of the rules. We have shown that this less complex strategy obtained groups of genes that scored comparably well in relation to previous studies, both in terms of ontology annotations and binding verification. In the current study we present two significant improvements of the above approach: using local expression similarity in clustering of time series data and extending the framework to include genes for which no expression profile data have been reported.

We have also attempted a comprehensive comparison of all of the above mentioned computational methods to assess their biological significance. This is not an easy task since no definitive source of genome-wide data on true co-regulation exists. Beer et al. [24] evaluated their ability to predict genes belonging to expression clusters using the classical training-test set division of the genes in a cross validation setting. However, there is of course no guarantee that these clusters correspond to truly co-regulated genes. Furthermore, the studies we compare applied different clustering methods resulting in different clusterings. Consequently, there is no consistent way of deciding whether a prediction is correct across different studies, and hence it seems inappropriate to use the ability to predict expression as an evaluation criterion here. Instead, we argue that a better approach for evaluating the predicted co-regulated genes is to apply external data/knowledge not used to induce the models themselves.

Using experimental binding data and Gene Ontology annotations, we demonstrate improvements in both quality (Tables 1, 2, 3) and coverage (Figure 4) when using local similarity of expression profiles. Furthermore, we showed that expanding the modules to include genes without known expression profiles did not reduce the quality of the rules and thus we conclude that the approach indeed is capable of generalizing to these genes.

Obviously, also the evaluation data used in this study may be associated with some disadvantages. The ChIP-Chip data is inevitably linked to a specific biological context, and thus different contexts/cellular states would result in different binding data and consequently different evaluation scores in our study. The use of functional categories to search for binding site motifs [8] may also provide an unfortunate bias when evaluating different methods using Gene Ontology. However, to the best of our knowledge, these are the most appropriate data sets for evaluation that exist. And by comparing different studies using the same evaluation method and the same data, any unfortunate bias should at least be the same for all the methods. On the one hand, the relatively large range of performance observed for the methods we have compared indicates that the problem of finding binding site modules is far from solved. On the other hand, it is also an indication that at least some non-trivial correlations between TF binding and sequence motifs or between functional annotation and sequence motifs are being picked up by our approach. Finally, the use of different data in terms of experimental data (TF binding), and human-curated knowledge (Gene Ontology) strengthens our confidence in the biological significance of the presented evaluations.

Similar methodology, i.e. IF-THEN rules connecting TF modules with expression profiles, was used in a recently published study of Pham et al. [30]. However, one difference is that we do not use Chromatin-Immunoprecipitation data [23,31] for rule inference but only as an independent standard for method evaluation. This means that our method is also applicable to organisms for which no genome-wide ChIP-Chip results are available. The underlying difference in approach also prevents us from direct comparisons with the results of that study. It should be mentioned here that although the rule induction method is applicable to all types of expression datasets, the particular time window improvement evaluated in this study obviously requires time profile expression data. However, the idea of measuring expression similarity over subsets of conditions could still be fruitful in non-time profile expression data by using so-called bi-clustering to identify subsets of conditions [32].

Results of all the performed analyses (i.e. using both statistical significance and pairwise co-regulation coefficient calculations) support the conclusion that local correlation of expression time profiles combined with sequence motif information leads to more accurate predictions of binding site modules than was possible with other methods. It is also clear, provided that enough data are available to develop the initial modules, that the method generalizes well over the genes for which no expression data are available.

## Methods

### Gene clustering using local similarity of expression

For clustering the expression profiles, we have adopted the procedure successfully employed in our earlier work [4]. However, two modifications were necessary:

• We have changed the distance function from Euclidean distance to Pearson correlation, since it is better suited for comparison of partial expression profiles.

• Since we are calculating the clustering for all possible time windows of sizes 4–8, we cannot afford to compute the clusters centered on every possible gene. Instead we iteratively exclude genes already assigned to a cluster from further processing. Although this makes the clustering potentially sensitive to the ordering of the genes, it is a necessary sacrifice to reduce the number of decision tables from which the rules are induced. Moreover, experiments with randomly reshuffled data have convinced us that, although different ordering of genes provides slightly different clusterings, it has no significant impact on the rules we obtain from the entire procedure.

The gene clustering algorithm allows for adjustable size and position of time windows, as well as adjustable correlation thresholds. Once those parameters are set, the algorithm starts with all genes and repeatedly

a) selects the first unassigned gene as a representative for the new cluster,

b) assigns all genes fulfilling the two criteria below to the newly created cluster:

(i) the Pearson correlation coefficient calculated with respect to the representative gene is above the selected threshold

(ii) comparison is restricted to the time window of the specified size and starting point.

### Rule induction

The procedure of rule induction is based on our previous approach [4] and modified as described below. Given a cluster of genes with expression correlated over a period of time we view the induction of rules as an instance of the classic decision problem. The set of all genes in the system constitutes the universe where the decision class corresponds to the membership in the cluster, and where the gene attributes are formed by a Boolean vector containing information on the presence or absence of each motif in the promoter. Given a decision table of this kind we can ask if there exists a combination of motifs allowing us to discern between the genes in the cluster and all other genes. Finding such combinations is NP-complete [33], so in Hvidsten et al. [4] we have employed the Rosetta software [34] based on the rough-set paradigm [35,36], which uses some well-established heuristics to obtain a set of locally optimal results. The set of rules inferred by the Rosetta system is then filtered in order to retain only the rules that are both general enough (i.e. cover more than 5 genes) and sufficiently accurate (i.e. at least 2/3 of the genes matching the LHS of the rule must be present in the cluster).

In the case of the *S. cerevisae *genome, it was first observed by Lee et al. [31] that the number of transcription factor binding sites present in one promoter is on average very low (in our dataset it is approximately 6). We therefore assume that the number of motifs to be considered for each cluster is small enough to successfully employ an exhaustive search yielding all possible results. Since we ignore the ordering and the number of motifs in the promoter, we consider all TF motifs to be symbols of a finite alphabet and treat binding site modules as words over this alphabet. For each gene set considered we construct a suffix tree including all words (sets of motifs) occurring in the promoters of genes in the set. We can quickly annotate each branch of that tree with the number of occurrences of the corresponding word. With this technique, we can safely avoid visiting the tree branches with coverage lower than required and eliminate unnecessary computations. By using an exact and exhaustive algorithm instead of a heuristic, we have obtained a broader set of modules with better quality. The development of the software dedicated for this task significantly increased the efficiency of rule induction.

### Data material

*S. cerevisae *cell cycle expression time series data were taken from Cho et al. [29]. The dataset contains expression profiles of 6601 genes at 17 time points, of which 2501 were selected for our analysis according to the following criteria:

• no missing values were allowed at any of the time points,

• divergently transcribed genes were excluded (i.e. two genes sharing the same promoter region).

Motif dataset was taken from Hughes et al. [8]. It consists of the occurrences of 47 binding site motifs in 5652 promoters found by the AlignACE motif finding program.

For evaluation with experimentally observed binding we used data from ChIP-Chip experiments published by Harbison et al. [23]. It contains evidence of binding for 352 transcription factors at 6229 intergenic regions. We take only binding occurrences reporting a p-value < 0.01 [23]. We have also performed the same evaluation using an earlier dataset [31] (with very similar results) and the results can be found on our supplementary materials website.

For Gene Ontology evaluation, we used the newest (as of April 2005) GO-DAG and annotations: GO version 1.419 and annotations Revision: 1.1109. All above-mentioned GO data can be downloaded from [37].

### Measuring statistical significance

To measure the statistical significance of our rules we use Bonferroni corrected p-values. Let us assume that we have a rule *R *and a family of gene sets ℱ
 MathType@MTEF@5@5@+=feaafiart1ev1aaatCvAUfKttLearuWrP9MDH5MBPbIqV92AaeXatLxBI9gBamrtHrhAL1wy0L2yHvtyaeHbnfgDOvwBHrxAJfwnaebbnrfifHhDYfgasaacH8akY=wiFfYdH8Gipec8Eeeu0xXdbba9frFj0=OqFfea0dXdd9vqai=hGuQ8kuc9pgc9s8qqaq=dirpe0xb9q8qiLsFr0=vr0=vr0dc8meaabaqaciaacaGaaeqabaWaaeGaeaaakeaaimaacqWFXeIraaa@3787@ constituting our reference (i.e. sets of genes bound by the same transcription factors or sets of genes sharing common annotations in Gene Ontology). For every rule *R*, we consider its overlap with all sets *f *∈ ℱ
 MathType@MTEF@5@5@+=feaafiart1ev1aaatCvAUfKttLearuWrP9MDH5MBPbIqV92AaeXatLxBI9gBamrtHrhAL1wy0L2yHvtyaeHbnfgDOvwBHrxAJfwnaebbnrfifHhDYfgasaacH8akY=wiFfYdH8Gipec8Eeeu0xXdbba9frFj0=OqFfea0dXdd9vqai=hGuQ8kuc9pgc9s8qqaq=dirpe0xb9q8qiLsFr0=vr0=vr0dc8meaabaqaciaacaGaaeqabaWaaeGaeaaakeaaimaacqWFXeIraaa@3787@ i.e. the set of genes from *f *matching the left-hand-side (LHS) of *R*. Then the p-value we calculate for the rule *R *and the set *f *is the probability of obtaining such overlap by chance, which can be computed from the hyper-geometric distribution:

p(x,N,n,k)=∑i=xmin(k,n)(ki)(N−kn−i)(Nn),
 MathType@MTEF@5@5@+=feaafiart1ev1aaatCvAUfKttLearuWrP9MDH5MBPbIqV92AaeXatLxBI9gBaebbnrfifHhDYfgasaacH8akY=wiFfYdH8Gipec8Eeeu0xXdbba9frFj0=OqFfea0dXdd9vqai=hGuQ8kuc9pgc9s8qqaq=dirpe0xb9q8qiLsFr0=vr0=vr0dc8meaabaqaciaacaGaaeqabaqabeGadaaakeaacqWGWbaCcqGGOaakcqWG4baEcqGGSaalcqWGobGtcqGGSaalcqWGUbGBcqGGSaalcqWGRbWAcqGGPaqkcqGH9aqpdaaeWbqaamaalaaabaWaaeWaaeaafaqabeGabaaabaGaem4AaSgabaGaemyAaKgaaaGaayjkaiaawMcaamaabmaabaqbaeqabiqaaaqaaiabd6eaojabgkHiTiabdUgaRbqaaiabd6gaUjabgkHiTiabdMgaPbaaaiaawIcacaGLPaaaaeaadaqadaqaauaabeqaceaaaeaacqWGobGtaeaacqWGUbGBaaaacaGLOaGaayzkaaaaaaWcbaGaemyAaKMaeyypa0JaemiEaGhabaacbiGae8xBa0Mae8xAaKMae8NBa4MaeiikaGIaem4AaSMaeiilaWIaemOBa4MaeiykaKcaniabggHiLdGccqGGSaalaaa@5A81@

where *x *is the size of the overlap, *N *is the number of all genes, *n *is the number of genes matching the left-hand side of the rule *R *and *k *= |*f*|.

For each rule we calculate a p-value against many sets (i.e. annotations, transcription factors). Since we only require that each rule has a low p-value for one of the sets, we apply the Bonferroni correction, which is a standard procedure to account for testing multiple hypotheses. We state that a rule *R *covering *n *out of *N *genes is statistically significant against ℱ
 MathType@MTEF@5@5@+=feaafiart1ev1aaatCvAUfKttLearuWrP9MDH5MBPbIqV92AaeXatLxBI9gBamrtHrhAL1wy0L2yHvtyaeHbnfgDOvwBHrxAJfwnaebbnrfifHhDYfgasaacH8akY=wiFfYdH8Gipec8Eeeu0xXdbba9frFj0=OqFfea0dXdd9vqai=hGuQ8kuc9pgc9s8qqaq=dirpe0xb9q8qiLsFr0=vr0=vr0dc8meaabaqaciaacaGaaeqabaWaaeGaeaaakeaaimaacqWFXeIraaa@3787@ if it has a Bonferroni corrected p-value of less than 0.01, i.e.

∃f∈ℱp(x(R,f),N,n,|f|)∗|ℱ|<0.01,
 MathType@MTEF@5@5@+=feaafiart1ev1aaatCvAUfKttLearuWrP9MDH5MBPbIqV92AaeXatLxBI9gBamrtHrhAL1wy0L2yHvtyaeHbnfgDOvwBHrxAJfwnaebbnrfifHhDYfgasaacH8akY=wiFfYdH8Gipec8Eeeu0xXdbba9frFj0=OqFfea0dXdd9vqai=hGuQ8kuc9pgc9s8qqaq=dirpe0xb9q8qiLsFr0=vr0=vr0dc8meaabaqaciaacaGaaeqabaWaaeGaeaaakeaacqGHdicjdaWgaaWcbaGaemOzayMaeyicI4mcdaGae8xmHyeabeaakiabdchaWjabcIcaOiabdIha4jabcIcaOiabdkfasjabcYcaSiabdAgaMjabcMcaPiabcYcaSiabd6eaojabcYcaSiabd6gaUjabcYcaSiabcYha8jabdAgaMjabcYha8jabcMcaPiabgEHiQiabcYha8jab=ftigjabcYha8jabgYda8iabicdaWiabc6caUiabicdaWiabigdaXiabcYcaSaaa@5932@

where *x*(*R*, *f*) calculates the number of genes from the set *f *covered by the left-hand side of the rule *R*. The Bonferroni correction only accounts for the multiple hypotheses tested for each individual rule. One could in addition include a similar correction for the fact that we induce a relativity large number of rules. However, here we opt for reporting the fraction of significant rules, which in all cases is much larger than what would be expected by chance (i.e. at the significance level 0.01 one would expect only 1% of the rules to be significant by chance).

In the case of Gene Ontology evaluation, for each rule we consider each part of the ontology separately (molecular function, cellular component, and biological process), and define ℱ
 MathType@MTEF@5@5@+=feaafiart1ev1aaatCvAUfKttLearuWrP9MDH5MBPbIqV92AaeXatLxBI9gBamrtHrhAL1wy0L2yHvtyaeHbnfgDOvwBHrxAJfwnaebbnrfifHhDYfgasaacH8akY=wiFfYdH8Gipec8Eeeu0xXdbba9frFj0=OqFfea0dXdd9vqai=hGuQ8kuc9pgc9s8qqaq=dirpe0xb9q8qiLsFr0=vr0=vr0dc8meaabaqaciaacaGaaeqabaWaaeGaeaaakeaaimaacqWFXeIraaa@3787@ as all annotations in that part of the ontology. In the case of ChIP-Chip data, we first consider all the TFs bound to any of the genes matching the rule, and then define ℱ
 MathType@MTEF@5@5@+=feaafiart1ev1aaatCvAUfKttLearuWrP9MDH5MBPbIqV92AaeXatLxBI9gBamrtHrhAL1wy0L2yHvtyaeHbnfgDOvwBHrxAJfwnaebbnrfifHhDYfgasaacH8akY=wiFfYdH8Gipec8Eeeu0xXdbba9frFj0=OqFfea0dXdd9vqai=hGuQ8kuc9pgc9s8qqaq=dirpe0xb9q8qiLsFr0=vr0=vr0dc8meaabaqaciaacaGaaeqabaWaaeGaeaaakeaaimaacqWFXeIraaa@3787@ as all the sets of genes bound by each of those transcription factors. For Gene Ontology, we copied all annotations made for any gene matching the rule upwards to all more general nodes in the ontology. We then considered ℱ
 MathType@MTEF@5@5@+=feaafiart1ev1aaatCvAUfKttLearuWrP9MDH5MBPbIqV92AaeXatLxBI9gBamrtHrhAL1wy0L2yHvtyaeHbnfgDOvwBHrxAJfwnaebbnrfifHhDYfgasaacH8akY=wiFfYdH8Gipec8Eeeu0xXdbba9frFj0=OqFfea0dXdd9vqai=hGuQ8kuc9pgc9s8qqaq=dirpe0xb9q8qiLsFr0=vr0=vr0dc8meaabaqaciaacaGaaeqabaWaaeGaeaaakeaaimaacqWFXeIraaa@3787@ to be all sets of genes annotated by any of these nodes.

### Supplementary data

Supplementary information for this article (full set of rules, additional charts and tables) can be found on our website [38]. We have also made a web interface for querying rules induced from the *S. cerevisae *cell-cycle. Through this interface one can search for rules including any particular gene or binding site.

## Authors' contributions

BW, TH, and KF conceptualized the project. BW implemented the local similarity clustering and rule inference framework, performed computations and wrote the original draft of the manuscript. TH developed and performed evaluations using Gene Ontology. All authors participated in the design of the study and contributed with critical remarks and ideas to improve the content of the manuscript.
